# The use of carer assisted adherence therapy for people with Parkinson's disease and their carers (CAAT-PARK): study protocol for a randomised controlled trial

**DOI:** 10.1186/1745-6215-12-251

**Published:** 2011-11-28

**Authors:** David J Daley, Katherine HO Deane, Richard J Gray, Paul F Worth, Allan B Clark, Kanagasabesan Sabanathan, Michael Pfeil, Phyo K Myint

**Affiliations:** 1 Faculty of Medicine and Health Sciences, University of East Anglia, Norwich Research Park, Norwich, Norfolk, NR4 7TJ, UK; 2 Norfolk & Norwich University Hospital NHS Foundation Trust, Colney Lane, Norwich, Norfolk, NR4 7UY, UK

**Keywords:** Parkinson's disease, Medication, Adherence, Adherence Therapy

## Abstract

**Background:**

Pharmacological intervention is essential for managing the symptoms of Parkinson's disease. Adherence to medication regimens however is a major problem. Poor adherence leads to significant motor deterioration and inadequate symptom control. This results in poor quality of life. Whilst interventions to improve medication adherence have shown considerable benefit in other chronic conditions, the efficacy of such treatments in Parkinson's disease is less well researched. Many people with Parkinson's disease require substantial support from spouse/caregivers. This often extends to medication taking. Consequently, spouse/caregiver's support for timely medication management is paramount. We aim to investigate the benefit of a novel intervention, Carer Assisted Adherence Therapy, for improving medication adherence and quality of life in people with Parkinson's disease. Adherence therapy may help to optimise the efficacy of anti-parkinsonian agents, subsequently improving clinical outcomes.

**Methods/Design:**

A parallel, randomised controlled trial will be conducted to investigate whether carer assisted adherence therapy is effective for improving medication adherence and quality of life. We aim to recruit 40 patient/carer pairs into each group. Participants will be randomly assigned by the Clinical Research Trials Unit at the University of East Anglia. Adherence therapy is a brief cognitive-behavioural approach aimed at facilitating a process of shared decision making. The central theory is that when patients make shared choices with a professional they are more likely to continue with those choices because they are personally owned and meaningful. Outcomes will be rates of adherence and quality of life, determined by the Morisky Medication Adherence Scale-4 and the Parkinson's disease Questionnaire-39 respectively. Assessments will take place post randomisation, immediately post intervention and 12-weeks post randomisation. Primary outcomes are adherence and quality of life at 12-week follow-up. Efficacy will be determined using intention-to-treat analysis. Independent samples t-tests will compare mean changes between groups from baseline to follow-up. Per protocol analysis will be conducted based on individuals with no major protocol deviation. Where imbalances in baseline characteristics are identified, an adjusted analysis will be performed using a regression model. Analysis will be masked to treatment allocation.

**Trial Registration:**

ISRCTN: ISRCTN07830951

## Background

### Parkinson's disease

Parkinson's disease (PD) is a progressive, disabling, neurodegenerative disease that significantly reduces quality of life (QoL) [1,2]. Debilitating symptoms of bradykinesia (slowness of movement), resting tremor, rigidity and postural instability are principal features of PD [1,3]. In addition to these motor symptoms, non-motor symptoms (NMS) such as cognitive impairment, dementia, sleep disturbances, depression and falls are significantly associated with reduced QoL [4]. Cognitive impairment is reported to affect 20-30% of patients with PD, even in the early stages of the disease [5]. As PD progresses cognitive decline persists and patients may develop dementia [6]. The cumulative prevalence has been reported to be substantial; at least 75% of people with PD who survive longer than 10 years will develop dementia [6]. As motor and NMS have considerable impact on QoL in PD, addressing both is therefore an integral part of management.

### Treatment & Regimen Complexity

The pharmacological management of PD is complex. Monoamine Oxidase-B (MAO-B) inhibitors, dopamine receptor agonists and Levodopa represent first line treatment options [1]. Typically younger individuals are treated with a MAO-B inhibitor (one daily dose), especially if symptoms are mild, or a dopamine receptor agonist (three daily doses) as first line intervention. Older (≥ 75 years) individuals, especially those with or at risk of cognitive impairment, may be treated with Levodopa as first line therapy [7,8]. Although management in early disease is usually adequate with monotherapy [7,8], more than half of people with PD take two to four anti-parkinsonian medications three to four times daily [9,10]. This is because multiple drug classes are warranted as PD progresses [11-14]. Furthermore, each drug may have different dosing schedules, further complicating regimens [9]. Catechol-O-Methyltransferase inhibitors can supplement Levodopa adding further complexity. Increasing doses and/or dosage frequency may also be required to adequately manage worsening symptoms in advanced stages [14]. With advancing disease, the therapeutic window narrows and becomes reliant on more frequent and specific interval dosing to maintain adequate treatment effect and avoid motor fluctuations [7,15]. Some people with advanced PD can take as many as ten doses a day in order to manage fluctuations [8,16]. Dyskinesias (involuntary movements) associated with long-term Levodopa use may also require remediation in later PD [16]. Additionally, specific non-motor complications necessitate further drug use [4,14].

### Medication Non-adherence

To achieve optimal symptom control, adherence to medication in PD is paramount [17]. However, it has been reported that a third to half of all medicines prescribed to people with long-term conditions are not taken as recommended [18-20]. Therefore, not surprisingly medication adherence is poor in people with PD, especially as symptoms of cognitive impairment and anxiety and depression have been reported to be highly prevalent and which impact negatively on medication adherence in this patient population [21]. Reported medication adherence in PD was as low as 10% in one study with 76% acknowledging miss-timed or missed doses [22]. For drugs requiring multiple daily doses, researchers reported only 3% fully adhered to medication regimens [23]. These findings suggest medication non-adherence is significant in people with PD. The consequences of non-adherence are substantial [24]. Poor adherence can result in wearing off of the treatment effect which can significantly increase motor dysfunction [15]. People with PD may also over-medicate with dopaminergic therapy [23]. This can result in severe dyskinesia, potentially lead to the development of impulse control disorder, especially when dopamine receptor agonists are used, and may even result in psychosis [25]. Dyskinesias have been associated with significant reductions in QoL [26].

### Carer Involvement

Although people may be completely independent in the early stages of PD, in advanced stages people usually require considerable support with daily activities [27]. Many people with PD receive support through informal carers such as a spouse or family member. This often extends to medication management, particularly aiding in the taking of medications [16,28]. For the caregiver, the responsibility for and help with timely management of a relative's anti-parkinsonian medication is essential [27].

### Study Rationale

There is a need for an intervention that enhances adherence to prescribed medication in PD. A targeted therapy is likely to be associated with overall improvement in rates of adherence, leading to optimal symptom control. Increasing motor fluctuations and dyskinesias resulting in disability have been associated with poor QoL [26]. As common motor symptoms of bradykinesia and rigidity are sensitive to anti-parkinsonian therapies [8], improved adherence to anti-parkinsonian medication may facilitate a mechanism for improving QoL.

Interventions aiming to improve adherence have demonstrated efficacy in other long-term conditions such as hypertension and psychotic disorders [29-32]. However, there is a paucity of evidence testing the efficacy of adherence interventions in PD. Previous research has identified a variety of factors that influence non-adherence in PD: problems with complex treatment regimens, polypharmacy, cognitive impairment and depressive symptoms [15,16,33,34]. Therefore, a patient centred therapy aimed at exploring personally relevant benefits of medication use, formulating problem solving strategies and exploring beliefs and concerns about medications may improve adherence to anti-parkinsonian drugs. It is also acknowledged that in PD caregiver involvement in fostering medication adherence can be substantial [16]. This is essential where significant cognitive impairment presents. Therefore, caregivers need to be supported in their role of encouraging medication adherence.

We hypothesise that an adherence therapy that targets both people with PD and their spouse/carer is likely to improve medication adherence and be associated with better QoL compared to treatment as usual.

## Methods/Design

### Trial Design

A parallel group, randomised controlled trial will be conducted to compare Carer Assisted Adherence Therapy (CAAT-PARK) with Treatment as Usual (TAU) for non-adherent people with PD and their spouse/carers. The study will compare the two groups immediately post intervention and at 12 weeks post randomisation (follow-up).

### Trial Objectives

To investigate whether people with PD and their spouses/carer who receive a programme of CAAT-PARK in addition to TAU show significantly greater rates of medication adherence and improved QoL from baseline to 12 week post randomisation compared to those who receive TAU only.

Secondary objectives are to investigate whether people who receive CAAT-PARK and those who receive TAU differ in terms of overall disease state, activities of daily living (ADL), beliefs about medication, generic health related QoL, and levels of carer distress. We will investigate whether baseline levels of cognitive impairment and anxiety and depression influence the effective uptake of CAAT-PARK. We also aim to investigate the experience of those receiving the intervention.

### Outcome Measures

#### Primary outcomes

• Change in adherence to medication determined by the Morisky Medication Adherence Scale (MMAS-4) [35]

• Change in QoL determined by the Parkinson's Disease Questionnaire-39 (PDQ-39) [36]

#### Secondary outcomes

People with PD:

• Movement Disorder Society - Unified Parkinson's Disease Rating Scale (MDS-UPDRS) Part I (non-motor experiences of daily living), Part II (motor experiences of daily living) and Part IV (motor complications) [37]

• Beliefs about Medication Questionnaire (BMQ) [38]

• EuroQol quality of life questionnaire (EQ-5D) [39]

Spouse/Carer Outcomes:

• Carer Distress Scale (CDS) [40]

• BMQ

### Trial Participants

People attending Medicine for the Elderly or Neurology outpatient appointments at a University Hospital in the East of England, UK, for diagnosed or probable PD. Spouse/carers will be invited to participate.

### Inclusion Criteria

1. Adults diagnosed with, or with probable, Idiopathic PD (three out of four of the chief UK Brain Bank criteria).

2. Prescribed one or more anti-parkinsonian medications by a Consultant Neurologist or Consultant Physician with specialist knowledge of movement disorders.

3. English speaking and literate (participants are required to actively engage in the therapy process).

4. Stable medication regime i.e. not altered within the previous month and not expected to change during the period of the research project (12 weeks).

5. Not demented or significantly cognitively impaired. The clinical team will judge whether the patient has the cognitive capacity required to participate fully in the trial i.e. read patient information, complete self report questionnaires and engage actively in the therapy process.

6. Show poor adherence as determined by a MMAS-4 score ≥ 2.

### Exclusion Criteria

1. Suspected Parkinsonism due to other causes than idiopathic PD.

2. Treated with anti-parkinsonian medications for a mental health complaint.

3. Diagnosed with dementia.

4. Life expectancy < 6 months.

### Recruitment

**Figure 1:**
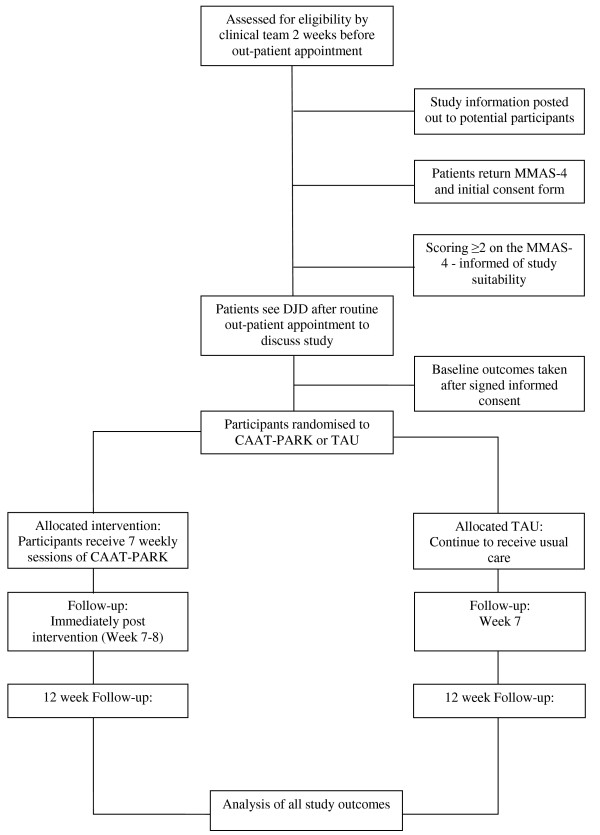
**CONSORT diagram - progression through CAAT-PARK**.

People who are potentially eligible based on inclusion criteria 1, 2, 3, 4 and 5 will be identified by the clinical team two weeks prior to upcoming outpatient appointments. An information pack containing: a patient invitation letter, participant information sheet, MMAS-4 and a consent form for the MMAS-4 will be posted out to those who are potentially eligible. This represents stage one consent i.e. willingness to consider study participation. An information sheet for the spouse/carer will supplement the patient information. Patients will be asked to return the MMAS-4 and the accompanying consent form. People scoring ≥ 2 will be informed by phone of their suitability to participate. Individuals who are willing to participate but do not meet eligibility criteria will be thanked for their interest. Eligible patients will be approached for their consent to participate in the trial on the day they attend clinic for their out-patient appointment (stage two consent). Recruitment will be a rolling programme over 12 - 14 months until recruitment targets are achieved. Figure 1 shows the CONSORT diagram for progression of participants throughout the study duration.

### Informed Consent

Thirty minutes will be set aside for taking informed consent to ensure patients/carers possess a sound understanding of the trial. At each point of participant contact ongoing verbal consent will be sought. If patients withdraw they will then receive standard care (TAU). Data collected to the point of withdrawal will be retained in the trial data set.

### Randomisation

Participants will be randomly assigned to CAAT-PARK or TAU. Randomisation will take place in a private room following signed informed consent and completion of baseline (pre-randomisation) measures. Randomisation will be completed using computer generated random numbers accessed via a web-based randomisation system developed by the Clinical Research Trials Unit (CRTU) at the University of East Anglia (UEA). Participants will be allocated a unique identifier number which will be sent to CRTU where allocation will be undertaken by permuted random blocks of four and six. Participants will be stratified into spouse/carer present or no spouse/carer present groups at randomisation respectively in order to investigate the potential effect modification of the spouse/carer on the treatment effect. The trial medical statistician (ABC) assisting in the analysis of all study data will be masked to participant group allocation.

### Baseline Assessments

Once the patient has consented some of the baseline data will be collected prior to randomisation. Baseline measures for primary outcomes (MMAS-4, PDQ-39) will be completed by participants at this stage to ensure data is acquired prior to randomisation. Participants will also be assessed in clinic prior to randomisation using measures that cannot be self-reported and require a rater, MDS-UPDRS parts I & IV and the Montreal Cognitive Assessment (MoCA). The Hospital Anxiety & Depression Scale (HADS) will also be completed at this stage. All scales will be completed in a private consultation room. Participants will be instructed how to complete all other baseline measures for secondary outcomes before taking them home to complete in their own time: BMQ, EQ-5D and MDS-UPDRS part II (patient questionnaire). Spouse/carers will take the baseline BMQ and CDS home to complete. Participants will be asked to return these self-reported questionnaires within two weeks of randomisation. The following information will be obtained:

### Description of Primary Measures

#### Morisky Medication Adherence Scale (MMAS-4)

The MMAS-4 is a self-report scale for identifying medication non-adherence [35] and has been used in PD [16,41]. The scale is constructed of four items answered by 'yes' or 'no'. Four 'no' responses signifies perfect adherence. Any 'yes' responses indicates some degree of non-adherence with medication. We aim to recruit people scoring two or more 'yes' responses.

#### Parkinson's Disease Questionnaire - 39 (PDQ-39)

The PDQ-39 is a PD-specific QoL questionnaire. The scale has been extensively tested for reliability and validity and is widely used in both research and clinical practice [42,43]. Items making up the scale measure eight dimensions of health which are related specifically to PD: mobility, ADL, emotional wellbeing, stigma, social support, cognition, communication and bodily discomfort.

### Description of Secondary Measures

#### Movement Disorder Society - Unified Parkinson's Disease Rating Scale (MDS-UPDRS)

The MDS-UPDRS is a revised version of the Unified Parkinson's Disease Rating Scale [37,44]. The MDS-UPDRS has four parts of which we will use three, namely, (I) Non-motor Experiences of Daily Living; (II) Motor Experiences of Daily Living; (IV) Motor Complications. This represents 32 items from the scale. Twenty questions are completed by the patient/caregiver as part of a questionnaire and 12 questions are completed by the assessor through a structured interview format - 6 items in part I and IV, respectively. When tested for its clinimetric properties, the scale has been shown to have high internal consistency, reliability and validity, and correlates well with the original UPDRS [44]. Competency based training and an online examination developed by the Movement Disorders Society have been completed by the Chief Investigator.

#### Beliefs about Medication Questionnaire (BMQ)

The BMQ is comprised of two scales: (1) an 11 item questionnaire relating to prescribed medication, (2) an 8 item questionnaire relating to general views about taking medication. The questionnaires assess beliefs about the necessity of prescribed medication for controlling illness and concerns about taking medications [38]. Respondents rate each item on a five point Likert-type scale depending on their degree of agreement (1 strongly disagree, 5 strongly agree). Higher scores indicate higher levels of concern or strong beliefs towards the use of medication.

#### EuroQoL (EQ-5D)

The EQ-5D is an established, standardised generic health utility index tool used extensively in clinical studies [39]. It consists of five items covering mobility, self-care, usual activity, pain/discomfort, and anxiety/depression domains. A visual analogue scale represents one final characteristic of the instrument. It provides a simple descriptive profile and can be used to estimate a single index value for a respondent's health status and change in Quality Adjusted Life Years (QALYs).

#### Caregiving Distress Scale (CDS)

The CDS is a concise measure designed to assess and profile informal caregivers with respect to stressful outcomes. The scale was developed in a PD population from various caregiving measures including a wide range of items and varying associations with distress [28,40]. The CDS comprises five distinct dimensions comprising 17 items that have a potential negative impact on caregivers. Answers are provided on a 0-4 scale based on level of agreement.

#### Satisfaction Questionnaire

To investigate patient and spouse/carer satisfaction with the CAAT-PARK process, we will post out a satisfaction questionnaire with the follow-up (week 12) outcome measures.

### Description of Other Measures (Prognostic Factors)

#### Montreal Cognitive Assessment Scale (MoCA)

The MoCA is a 30-point scale delivered by a rater. The MoCA covers a range of executive functions and has been proposed to be the most appropriate scale for assessing cognitive impairment in PD research where cognition is not the primary outcome [45]. The MoCA has six orientation questions and a five word memory recall task. A clock drawing task and a cube copy test assess visuospatial function. Attention/concentration is assessed using serial 7's, target mapping and digit span forward and backwards tasks. Executive functions are evaluated using a shortened version of the Trial Making B Test, phonemic fluency, and a verbal abstraction task.

#### Hospital Anxiety and Depression Scale (HADS)

The HADS is a self screening questionnaire for anxiety and depression [46]. The HADS consists of 14 questions, seven for each anxiety and depression and has been widely used and validated [47].

In addition to levels of anxiety/depression and cognitive capacity, we will identify and present demographics at baseline: participants' age, duration and severity of PD (Hoehn & Yahr score), medication profile (type of medications, dosage level and frequency), socioeconomic status, co-morbidities, level of education and whether medication is self-administered or given by a spouse/carer.

### Follow-up Measurements

**Figure 2:**
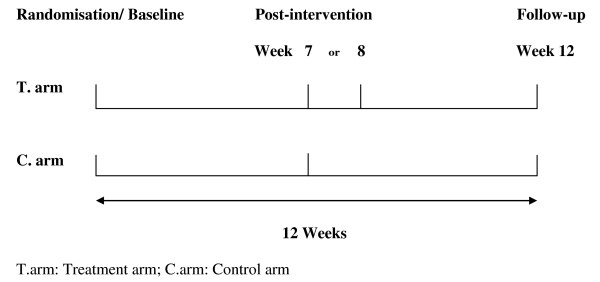
**Study Design - assessment time points**.

Baseline measures assessed to determine the efficacy of CAAT-PARK will be repeated immediately post intervention and at 12-weeks post randomisation (follow-up) (Figure 2). For the control group receiving TAU, assessments will be at week seven and week twelve. For the CAAT-PARK group, post intervention assessments will be at week seven or eight and then at week 12. This additional week at the midway post intervention time point accommodates potential sickness or time away providing flexibility from a pragmatic perspective.

## Intervention

### Description of Adherence Therapy

**Figure 3:**
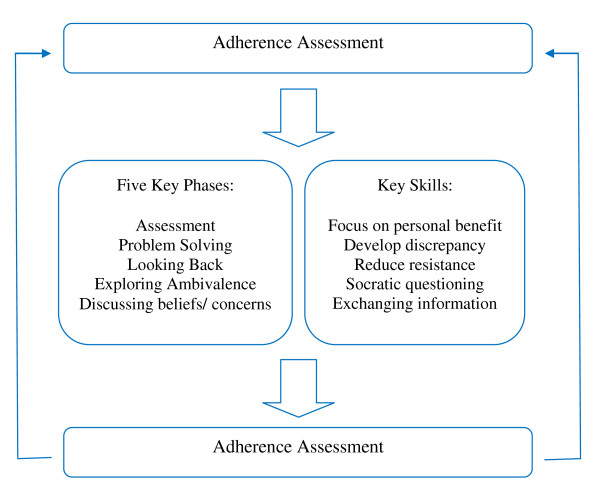
**CAAT-PARK model**.

CAAT-PARK is a brief, cognitive-behavioural approach aimed at facilitating a process of shared decision making [29,48]. CAAT-PARK is rooted in the observation that a person's beliefs impact on treatment adherence. The central theory is that when people make shared choices with a professional they are more likely to continue with those choices because they are personally owned and meaningful [30]. Identification and amplification of the personally relevant benefits of treatment, modifying beliefs about medication and exploring ambivalence towards medication taking behaviour represent interrelated constructs that are central tenants of the therapy. CAAT-PARK is delivered in five phases that form the core of the therapy (Figure 3): *assessment, medication problem-solving, a medication timeline (looking back), exploring ambivalence, and discussing beliefs and concerns about medication*. Key therapy skills incorporate exchanging information, developing discrepancy between the patient's thoughts and behaviours about medication, socratic style questioning and working with resistance to discussing medication and treatment. The aim of CAAT-PARK is to achieve a mutual, informed decision about medication between the individual and therapist. A key concept is that where patients and therapists make choices about treatment mutually, adherence to that regimen will be enhanced [48,49].

Participants allocated to CAAT-PARK will receive seven 30 minute sessions at weekly intervals. Assessment and problem solving will be delivered twice, making up seven sessions in total. Each weekly session will incorporate a separate theme, however, each session will be participant centred. Where a patient's carer has consented to the trial, the intervention will be delivered to the carer at the same time. Ten sessions over the course of the trial will be recorded to determine treatment fidelity against the CAAT-PARK manual [49].

### Treatment as Usual

Participants randomised to TAU will receive no additional information regarding medication adherence. Care will continue as usual according to routine practice. We will not provide any guidance to the clinical team as to the content of the usual care. Routinely usual care constitutes a clinic visit every 9-12 months to see the hospital consultant who is managing the patient's PD. Neurology specialist nurses may also see patients for routine follow-up between consultant clinics.

### Adverse Event Monitoring

Adverse events (AE) will be determined at each weekly visit and will commence from the point of randomisation up to week 12 follow-up. An AE checklist was developed following consensus views of the Trial Steering Committee (TSC) and medical specialists. AEs will be reported to the TSC and the participants' clinical team for appropriate action. All AEs will be addressed according to local Standard Operating Procedures (SOPs) for clinical trials of non-Investigational Medicinal Products (non-IMPs) developed in accordance with the Medicines for Human Use Regulations (2004) and the Department of Health's Research Governance Framework for Health and Social Care for identifying, recording, and reporting adverse events in clinical trials.

## Analysis

### Baseline Analyses

To assess external validity, demographic, and clinical characteristics of study participants', responses at baseline will be compared for participants who are subsequently randomised and participants who are screened but not randomised. The specific criteria by which participants are excluded from randomisation will be tabulated. Demographic and clinical characteristics will be compared between CAAT-PARK and TAU groups to identify potential imbalances not accounted for by randomisation.

### Efficacy Analysis of Primary Outcomes

The efficacy of CAAT-PARK will be determined by comparing the mean change in values of the two groups from baseline to week 12 follow-up. Analysis between the two groups will be made using an independent sample *t*-test. Intention to treat (ITT) and per protocol (PP) analyses will be undertaken. For the intervention group, this is irrespective of their compliance with CAAT-PARK. ITT analysis will represent the primary analysis and will be used for evaluation of all outcomes. PP analysis will include patients who comply with the treatment as defined by completing five or more out of seven CAAT-PARK sessions. All participants will undergo sub-group analysis testing for potential effect modification for the presence of a spouse/carer on the treatment effect. Appropriate adjustments will be made in the statistical analyses for potential factors that are unevenly distributed between the groups. Specifically, participants with significantly high reported levels of cognitive impairment and anxiety and depression at baseline (assessed with the MoCA and HADS, respectively) will be analysed to determine the independent impact of these prognostic factors on the treatment effect. Adjusted estimates will be obtained by identifying baseline imbalances and incorporating these into a regression model using pre and post intervention primary outcome scores.

If normal distribution assumptions are not met, even after suitable transformations, non-parametric analysis will be adopted. Imputation for missing/incomplete data will be carried out using chained equations with all outcome measures and potentially correlated baseline values. A total of 10 imputed datasets will be created.

### Sample Size

We will recruit a total of ninety-two family units (patient/carer pairs or patients alone), 46 per treatment group. This includes an additional 15% (n = 6) for potential participant attrition in each group. Where possible we aim to recruit patients with a spouse/carer, however, we will not exclude patient participants who do not have a spouse/carer but are wishing to participate. Using the primary outcomes, a pragmatic sample size of 40 participants per treatment group would provide an alpha of 0.05 and an 81% power to detect a difference of 25% improvement in medication adherence in the intervention group (detected by a one point shift in the MMAS-4) against 0% in the control group. This also provides 80% power to detect a Cohen's effect size of 0.69 in the PDQ-39, based on the published standard deviation of 8.89 in this patient group [43]. This would allow a difference in means of 6.13 (8.89 × 0.69) units in the PDQ-39.

### Qualitative Evaluation

Semi-structured interviews will be undertaken with a purposively selected sub-sample of patients (n = 10) and carers (n = 10) to explore the process and experience of receiving CAAT-PARK. Participants wishing to be interviewed will be pooled together and selected at random. This will ensure identification of participants wanting to be interviewed whilst preventing selection bias. Interviews will be thirty minutes in duration. The aims are to illuminate the quantitative findings by obtaining insight into the experience of receiving CAAT-PARK; consider which elements of CAAT-PARK are most helpful; explore perceptions of how CAAT-PARK has influenced medication taking; uncover potential barriers to receiving CAAT-PARK and explore how CAAT-PARK could be enhanced. All interview data will be audio recorded and transcribed verbatim. The transcripts will be coded using thematic analysis [50]. Analysis aims to ultimately inform the study outcome of patient acceptability of CAAT-PARK.

### Ethical Considerations

Although we do not anticipate any risk to study participants, we acknowledge the small possibility that increased medication adherence in PD may lead to greater incidence of dyskinesia. Dyskinesia is a common motor complication resulting from dopaminergic therapy in PD and will therefore be monitored by the TSC. It is theoretically possible that greater medication adherence may trigger drug induced psychosis, a rare but important complication of dopaminergic therapy in PD, so this will also be monitored in all participants receiving CAAT-PARK for its potential development.

The trial protocol and all related documents were given a favourable ethical opinion by Cambridge Central NHS Research Ethics Committee (REC reference number: 11/EE/0179).

## Discussion

Many people with PD do not take their anti-parkinsonian medications as prescribed. Sub-optimal adherence to prescribed medication in people with PD results in poor symptom control. This considerably impacts on QoL. Previous research has reported the benefit of various interventions including adherence therapy for improving medication adherence in other long-term conditions. Through the use of CAAT-PARK we aim to improve adherence behaviour in people with PD who are non-adherent to their prescribed medication. Furthermore, we aim to examine whether CAAT-PARK is effective for improving QoL in this patient group.

We have recently reported the effectiveness of adherence therapy in a trial of 136 hypertensive patients [51]. Improvements in medication adherence resulted in a substantial reduction in both systolic and diastolic blood pressures. Researchers have also tested the efficacy of other adherence intervention programmes in patients with psychotic disorders. One study showed people with psychotic symptoms in schizophrenia significantly improved after receiving a programme of adherence therapy [52]. Findings have shown similar adherence therapy interventions to provide direct benefit regarding service engagement and medication adherence [53]. Moreover, patient attitudes towards and satisfaction with medication improved in those who received treatment adherence therapy [53]. Such positive findings offer preliminary evidence of efficacy for the use of adherence interventions in people with long-term conditions and, furthermore, highlight patient acceptability of therapeutic adherence programmes.

Despite supportive findings, there is a paucity of randomised controlled trial evidence investigating the efficacy of interventions for improving medication adherence in people with PD. Findings from one study showed a 13% significant difference in timing adherence pre to post intervention between study groups after receiving simple didactic information relating to the continuous dopaminergic theory [54]. A recent study used a standardised eight week educational programme delivered to people with PD and their carers aiming to address psychosocial issues [55]. Findings showed an improvement in mood and less psychosocial problems and need for help in primary caregivers after receiving the intervention. Additionally patients improved in mood; however, there was only a trend towards significance in patients receiving the programme in relation to QoL [55].

The present study will use CAAT-PARK, a manualised intervention package that aims to ensure people with PD and their spouse/carers develop a vital understanding of the importance and personal benefit of sound medication adherence. Unlike previous studies, CAAT-PARK further recognises the importance of the spouse/carer for optimising medication adherence. Medication taking behaviour is a complex phenomenon in PD. A patient with PD may stop, omit doses, increase or decrease his or her medication based on the patient's perception of his or her own condition, the efficacy of the treatment, and his or her understanding of the indication for a particular drug, either accurate or fallacious. Discontinuing treatment secondary to actual or perceived adverse reactions, medication sparing (fear of becoming immune to treatment) and fear of long-term adverse effects of therapy have further been proposed [21]. Although didactic information has been shown to be effective we argue the importance of shared decision-making between patients and professionals, encouraging a dynamic and reflective process. With CAAT-PARK we attempt to identify and emphasise the personally relevant benefits to medication, which we argue will lead to positive behaviour change and resultantly improve medication adherence.

### Study Strengths

Unlike previous studies investigating adherence therapy programmes, we acknowledge the importance of the carer in assisting people with PD to take their medications as prescribed. Due to this dynamic, CAAT-PARK will be aimed at both the person with PD and their spouse/carer. The therapy will be delivered in the participants' homes by the same individual (DJD) who has received specific training in the delivery of CAAT-PARK. This has a binary affect in that it provides greater ecological validity and broadens access to the therapeutic intervention for severely affected PD patients. Furthermore, the delivery of CAAT-PARK by one trained clinician eradicates proficiency bias and ensures standardisation and consistency in the administration of the intervention. Although eradication of proficiency bias may be at the expense of high internal validity, we have shown the therapy to be effective in other long-term conditions using various trained clinicians. We therefore have no reason to believe the therapeutic intervention lacks validity regarding its delivery.

### Study Limitations

The subjective nature of the self report instruments used for evaluation of CAAT-PARK is acknowledged and every effort will be made to minimise this potential bias. In particular, patients may over or under report their true health status depending on the trial arm to which they are assigned. Baseline primary outcome measures will be completed prior to randomisation in attempt to reduce this bias. Resulting from the one-to-one participatory nature of CAAT-PARK, it will not be possible to mask study participants to their group allocation. Therefore, secondary outcomes at baseline (completed post randomisation) and all follow-up study outcomes will not be blinded.

### Prognostic Factors

Data from studies have shown cognitive function and mood to influence medication adherence [56,57]. It has been previously reported that depressed patients are considerably more likely to adhere poorly to medication regimens than non-depressed patients [58]. Depression has also been shown to negatively affect adherence rates in PD [23,34]. Cognitive impairment represents a major risk factor for non-adherence in people with PD [14,59]. Cognitive impairment has further been associated with under and over-use of medication in PD [14,45,57]. Therefore, it is possible that both cognitive impairment and the presence of anxiety and depression may deleteriously influence the effective uptake of the adherence intervention. We therefore aim to assess for these prognostic factors at baseline. Data collected for participants with identified cognitive impairment (assessed using the MoCA) or anxiety and depression (assessed using the HADS) will be appropriately adjusted using regression modelling. Where cognitive impairment and anxiety and depression are present in study participants at baseline assessment, analyses will be undertaken to assess their independent impact on the efficacy of CAAT-PARK. Further, we will examine which aspects of the MoCA, if any, predict poor medication adherence in the trial participants.

## Conclusion

Addressing issues surrounding non-adherence by problem solving and discussing concerns about medication may facilitate optimal symptom management in PD through greater medication adherence. If CAAT-PARK demonstrates efficacy for improving medication adherence and QoL in people with PD and their spouse/carers, health professionals will be able to incorporate the intervention into their routine practice with minimal training. As this has yet to be investigated, we intend to establish the efficacy of this novel intervention for people with PD and their spouse/carers in this RCT. This study will provide new knowledge about the effectiveness of optimising the efficacy of anti-parkinsonian agents using a targeted carer assisted adherence therapy in PD.

## Trial Status

Ongoing. At the time of submission patient recruitment was 16% of the overall target eight weeks post trial commencement. Recruitment rates are on target and will continue as a rolling process for the full study period.

## List of Abbreviations

PD: Parkinson's disease; QoL: Quality of Life; AT: Adherence Therapy; CAAT-PARK: Carer Assisted Adherence Therapy for people with Parkinson's disease; NMS: Non-Motor Symptoms; TAU: Treatment As Usual; MMAS: Morisky Medication Adherence Scale-4; PDQ-39: Parkinson's Disease Questionnaire-39; MDS-UPDRS: Movement Disorder Society - Unified Parkinson's Disease Rating Scale; BMQ: Beliefs about Medication Questionnaire; EQ-5D: EuroQol; QALYs: Quality Adjusted Life Years; CDS: Carer Distress Scale; CRTU: Clinical Research Trials Unit; UEA: University of East Anglia; ADL: Activities of Daily Living; MoCA: Montreal Cognitive Assessment Scale; HADS: Hospital Anxiety and Depression Scale; AE: Adverse Events; non-IMP's: non-Investigational Medicinal Products; TSC: Trial Steering Committee; GCP: Good Clinical Practice; ITT: Intention to Treat; PP: Per Protocol; SOP's: Standard Operating Procedures.

## Competing interests

The authors declare that they have no competing interests.

## Authors' contributions

DJD, PKM, KHOD, RJG, ABC, PW and KS all contributed to the design of the study. MP contributed to the protocol for qualitative data analysis. DJD, PKM, KHOD, RJG and PW drafted the paper. All authors provided revisions of the paper and have approved the final manuscript.

## References

[B1] NICE The National Collaborating Centre for Chronic Conditions. Parkinson's Disease: National clinical guideline for diagnosis and management in primary and secondary care 2006 London: Royal College of Physicians 21089238

[B2] Schapira AHV Obeso J Timing of treatment initiation in Parkinson's disease: A need for reappraisal? Ann Neurol 2006 59 3 559 562 10.1002/ana.20789 16489611

[B3] Albanese A Diagnostic criteria for Parkinson's disease Neurological Sciences 2003 24 0 s23 s26 10.1007/s100720300032 12774207

[B4] Chaudhuri KR Healy DG Schapira AHV Non-motor symptoms of Parkinson's disease: diagnosis and management The Lancet Neurology 2006 5 3 235 245 10.1016/S1474-4422(06)70373-8 16488379

[B5] Aarsland D Zaccai J Brayne C A systematic review of prevalence studies of dementia in Parkinson's disease Movement Disorders 2005 20 10 1255 1263 10.1002/mds.20527 16041803

[B6] Aarsland D Kurz MW The Epidemiology of Dementia Associated with Parkinson's Disease Brain Pathology 2010 20 3 633 639 10.1111/j.1750-3639.2009.00369.x 20522088 PMC8094858

[B7] Schapira AHV Emre M Jenner P Poewe W Levodopa in the treatment of Parkinson's disease European Journal of Neurology 2009 16 9 982 989 10.1111/j.1468-1331.2009.02697.x 19538218

[B8] Schapira AHV Treatment Options in the Modern Management of Parkinson Disease Arch Neurol 2007 64 8 1083 1088 10.1001/archneur.64.8.1083 17698697

[B9] Leoni O Martignoni E Cosentino M Michielotto D Calandrella D Zangaglia R Riboldazzi G Oria C Lecchini S Nappi G Drug prescribing patterns in Parkinson's disease: a pharmacoepidemiological survey in a cohort of ambulatory patients Pharmacoepidemiology and Drug Safety 2002 11 2 149 157 10.1002/pds.682 11998540

[B10] Tan EK Yeo AP Tan V Pavanni R Wong MC Prescribing pattern in Parkinson's disease: are cost and efficacy overriding factors? Int J Clin Pract 2005 59 5 511 514 10.1111/j.1368-5031.2005.00426.x 15857344

[B11] Schapira A Agid Y Barone P Jenner P Lemke M Poewe W Rascol O Reichmann H Tolosa E Perspectives on recent advances in the understanding and treatment of Parkinson's disease European Journal of Neurology 2009 16 10 1090 1099 10.1111/j.1468-1331.2009.02793.x 19723294

[B12] Holloway RG Shoulson I Fahn S Kieburtz K Lang A Marek K McDermott M Seibyl J Weiner W Musch B Pramipexole vs levodopa as initial treatment for Parkinson disease: a 4-year randomized controlled trial Arch Neurol 2004 61 7 1044 1053 15262734 10.1001/archneur.61.7.1044

[B13] Rascol O Brooks DJ Korczyn AD De Deyn PP Clarke CE Lang AE A Five-Year Study of the Incidence of Dyskinesia in Patients with Early Parkinson's Disease Who Were Treated with Ropinirole or Levodopa New England Journal of Medicine 2000 342 20 1484 1491 10.1056/NEJM200005183422004 10816186

[B14] Bainbridge JL Ruscin J Challenges of Treatment Adherence in Older Patients with Parkinson's Disease Drugs Aging 2009 26 2 145 155 10.2165/0002512-200926020-00006 19220071

[B15] Grosset KA Reid JL Grosset DG Medicine-taking behavior: Implications of suboptimal compliance in Parkinson's disease Movement Disorders 2005 20 11 1397 1404 10.1002/mds.20525 16092116

[B16] Valldeoriola F Coronell C Pont C Buongiorno MT Cámara A Gaig C Compta Y the members of the ASG Socio-demographic and clinical factors influencing the adherence to treatment in Parkinson's disease: the ADHESON study European Journal of Neurology 2010 no-no 10.1111/j.1468-1331.2010.03320.x 21199185

[B17] Rigby D Adherence assessment tools: Drugs dont work when they're not taken The Australian Journal of Pharmacy 2007 88 32 33

[B18] Haynes RB McDonald HP Garg AX Helping Patients Follow Prescribed Treatment JAMA: The Journal of the American Medical Association 2002 288 22 2880 2883 10.1001/jama.288.22.2880 12472330

[B19] NICE Medicines Adherence: Involving patients in decisions about prescribed medications and supporting adherence. NICE Clinical Guidelines 76 Developed by the National Collaborative Centre for Primary Care 2009

[B20] Osterberg L Blaschke T Adherence to Medication New England Journal of Medicine 2005 353 5 487 497 10.1056/NEJMra050100 16079372

[B21] Grosset D European PDTCSG Therapy adherence issues in Parkinson's disease Journal of the Neurological Sciences 2010 289 1-2 115 118 10.1016/j.jns.2009.08.053 19793590

[B22] Leopold NA Polansky M Hurka MR Drug adherence in Parkinson's disease Movement Disorders 2004 19 5 513 517 10.1002/mds.20041 15133814

[B23] Grosset KA Bone I Grosset DG Suboptimal medication adherence in Parkinson's disease Movement Disorders 2005 20 11 1502 1507 10.1002/mds.20602 16037924

[B24] Chaudhuri K Taurah L MacMahon D PD LIFE: a prospective multi-centre longitudinal audit of quality of life in Parkinson's disease across the UK J Neurol Neurosurg Psychiatry 2004 75 516

[B25] O'Sullivan SS Evans AH Lees AJ Dopamine dysregulation syndrome: an overview of its epidemiology, mechanisms and management CNS Drugs 2009 23 2 157 170 10.2165/00023210-200923020-00005 19173374

[B26] Péchevis M Clarke CE Vieregge P Khoshnood B Deschaseaux-Voinet C Berdeaux G Ziegler M the Trial Study G Effects of dyskinesias in Parkinson's disease on quality of life and health-related costs: a prospective European study European Journal of Neurology 2005 12 12 956 963 10.1111/j.1468-1331.2005.01096.x 16324089

[B27] A'Campo L Spliethoff-Kamminga N Macht M Roos R Caregiver education in Parkinson's disease: Formative evaluation of a standardized program in seven European countries Quality of Life Research: An International Journal of Quality of Life Aspects of Treatment, Care & Rehabilitation 2010 19 1 55 64 10.1007/s11136-009-9559-y 19946755 PMC2804793

[B28] Cifu D Carne W Brown R Pegg P Ong J Qutubuddin A Baron M Caregiver distress in parkinsonism Journal of Rehabilitation Research and Development 2006 43 4 499 508 10.1682/JRRD.2005.08.1365 17123189

[B29] Kemp R Kirov G Everitt B Haywood P David A Ramdomised Controlled Trial of Compliance Therapy: 18-month follow-up British Journal of Psychiatry 1998 172 413 10.1192/bjp.172.5.413 9747403

[B30] Gray R White J Schulz M Abderhalden C Enhancing medication adherence in people with schizophrenia: An international programme of research International Journal of Mental Health Nursing 2010 19 1 36 44 10.1111/j.1447-0349.2009.00649.x 20074202

[B31] Alhalaiqa F Deane K Nawafleh A Clark A Gray R Adherence therapy for medication non-compliant patients with hypertension: a randomised controlled trial Journal of Human Hypertension 2011 10.1038/jhh.2010.133 PMC3257548 21326328

[B32] Staring ABP Van der Gaag M Koopmans GT Selten JP Van Beveren JM Hengevald MW Loonen AJM Mulder CL Treatment adherence therapy in people with psychotic disorders: randomised controlled trial The British Journal of Psychiatry 2010 197 448 455 10.1192/bjp.bp.110.077289 21119150

[B33] Grosset D Antonini A Canesi M Pezzoli G Lee A Shaw K Cubo E Martinez-Martin P Rascol O Negre-Pages L Adherence to Antiparkinson Medication in a Multicenter European Study Movement Disorders 2009 24 6 826 832 10.1002/mds.22112 19191340

[B34] Evans AH Lawrence AD Potts J Appel S Lees AJ Factors influencing susceptibility to compulsive dopaminergic drug use in Parkinson disease Neurology 2005 65 10 1570 1574 10.1212/01.wnl.0000184487.72289.f0 16301483

[B35] Morisky D Green LW Levin DM Concurrent and Predictive Validity of a Self-reported Measure of Medication Adherence MEDICAL CARE 1986 24 1 67 74 10.1097/00005650-198601000-00007 3945130

[B36] Peto V Jenkinson C Fitzpatrick R Greenhall R The development and validation of a short measure of functioning and well being for individuals with Parkinson's disease Quality of Life Research 1995 4 3 241 248 10.1007/BF02260863 7613534

[B37] Goetz CG Fahn S Martinez-Martin P Poewe W Sampaio C Stebbins GT Stern MB Tilley BC Dodel R Dubois B Movement Disorder Society-sponsored revision of the Unified Parkinson's Disease Rating Scale (MDS-UPDRS): Process, format, and clinimetric testing plan Movement Disorders 2007 22 1 41 47 10.1002/mds.21198 17115387

[B38] Horne R Weinman J Hankins M The beliefs about medicines questionnaire: The development and evaluation of a new method for assessing the cognitive representation of medication Psychology & Health 1999 14 1 1 2422125612

[B39] Brooks R EuroQol: the current state of play Health policy 1996 37 1 53 72 10.1016/0168-8510(96)00822-6 10158943

[B40] Cousins R Davies AD Turnbull CJ Playfer JR Assessing caregiving distress: A conceptual analysis and a brief scale British Journal of Clinical Psychology 2002 41 4 387 403 10.1348/014466502760387506 12437793

[B41] Elm JJ Kamp C Tilley BC Guimaraes P Fraser D Deppen P Brocht A Weaver C Bennett S Investigators NN-P Self-reported adherence versus pill count in Parkinson's disease: the NET-PD experience Movement Disorders 2007 22 6 822 827 10.1002/mds.21409 17357141

[B42] Peto V Jenkinson C Fitzpatrick R PDQ-39: a review of the development, validation and application of a Parkinsopn's disease quality of life questionniare and its associated measures Journal of Neurology 1998 245 1 S10 S14 10.1007/PL00007730 9617716

[B43] Peto V Jenkinson C Fitzpatrick R Determining minimally important differences for the PDQ 39 Parkinson's disease questionnaire Age and Ageing 2001 30 4 299 10.1093/ageing/30.4.299 11509307

[B44] Goetz CG Tilley BC Shaftman SR Stebbins GT Fahn S Martinez-Martin P Poewe W Sampaio C Stern MB Dodel R Movement Disorder Society-sponsored revision of the Unified Parkinson's Disease Rating Scale (MDS-UPDRS): Scale presentation and clinimetric testing results Movement Disorders 2008 23 15 2129 2170 10.1002/mds.22340 19025984

[B45] Chou KL Amick MM Brandt J Camicioli R Frei K Gitelman D Goldman J Growdon J Hurtig HI Levin B A recommended scale for cognitive screening in clinical trials of Parkinson's disease Movement Disorders 2010 25 15 2501 2507 10.1002/mds.23362 20878991 PMC2978783

[B46] Zigmond AS Snaith RP The Hospital Anxiety and Depression Scale Acta Psychiatrica Scandinavica 1983 67 6 361 370 10.1111/j.1600-0447.1983.tb09716.x 6880820

[B47] Bjelland I Dahl AA Haug TT Neckelmann D The validity of the Hospital Anxiety and Depression Scale: An updated literature review Journal of psychosomatic research 2002 52 2 69 77 10.1016/S0022-3999(01)00296-3 11832252

[B48] Gray R Leese M Bindman J Becker T Burti L David A Gournay K Kikkert M Koeter M Puschner B Adherence Therapy for people with Schizophrenia. European multicentre randomised controlled trial British Journal of Psychiatry 2006 189 508 514 10.1192/bjp.bp.105.019489 17139034

[B49] Gray R Adherence Therapy: working together to improve health. A treatment manual for healthcare workers University of East Anglia 2011 http://eastanglia.academia.edu/RichardGray/Books/718181/Adherence_therapy_manual

[B50] Braun V Clarke V Using thematic analysis in psychology Qualitative Research in Psychology 2006 3 2 77 101 10.1191/1478088706qp063oa

[B51] Alhalaiqa F Deane KHO Nawafleh AH Clark A Gray R Adherence therapy for medication non-compliant patients with hypertension: a randomised controlled trial J Hum Hypertens 2011 10.1038/jhh.2010.133 PMC3257548 21326328

[B52] Maneesakorn S Robson D Gournay K Gray R An RCT of adherence therapy for people with schizophrenia in Chiang Mai, Thailand Journal of Clinical Nursing 2007 16 0 1302 1312 17584349 10.1111/j.1365-2702.2007.01786.x

[B53] Staring ABP Van der Gaag M Koopmans GT Selten JP Van Beveren JM Hengeveld MW Loonen AJM Mulder CL Treatment adherence therapy in people with psychotic disorders: randomised controlled trial The British Journal of Psychiatry 2010 197 6 448 455 10.1192/bjp.bp.110.077289 21119150

[B54] Grosset KA Grosset DG Effect of educational intervention on medication timing in Parkinson's disease: a randomized controlled trial BMC Neurology 2007 7 1 20 10.1186/1471-2377-7-20 17634109 PMC1931606

[B55] A'Campo LEI Wekking EM Spliethoff-Kamminga NGA Le Cessie S Roos RAC The benefits of a standardized patient education program for patients with Parkinson's disease and their caregivers Parkinsonism & related disorders 2010 16 2 89 95 10.1016/j.parkreldis.2009.07.009 19674927

[B56] Coons S Sheahan S Martin S Hendricks J Robbins C Johnson J Predictors of medication noncompliance in a sample of older adults Clinical Therapeutics 1994 16 1 110 8205597

[B57] MacLaughlin EJ Raehl CL Treadway AK Sterling TL Zoller DP Bond CA Assessing Medication Adherence in the Elderly: Which Tools to Use in Clinical Practice? Drugs &amp; Aging 2005 22 231 255 15813656 10.2165/00002512-200522030-00005

[B58] DiMatteo MR Lepper HS Croghan TW Depression Is a Risk Factor for Noncompliance With Medical Treatment: Meta-analysis of the Effects of Anxiety and Depression on Patient Adherence Arch Intern Med 2000 160 14 2101 2107 10.1001/archinte.160.14.2101 10904452

[B59] MacLaughlin EJ Raehl CL Treadway AK Sterling TL Zoller DP Bond CA Assessing Medication Adherence in the Elderly: Which tools to use in clinical practice? Drugs Aging 2005 22 3 231 255 10.2165/00002512-200522030-00005 15813656

